# Progress of rehabilitation in assisted living for mentally ill according to STAX-SA taxonomy

**DOI:** 10.1177/00207640241298902

**Published:** 2024-11-16

**Authors:** Erfan Jahangiri, Matti Viljakainen, Ann-Sofie Silvennoinen, Joel Ketola, Helinä Hakko, Pirkko Riipinen, Sami Räsänen

**Affiliations:** 1 Faculty of Medicine, Research Unit of Clinical Medicine, Psychiatry, University of Oulu, Finland; 2 Faculty of Education and Welfare Studies, Åbo Akademi University, Vasa, Finland; 3 Department of Psychiatry, Oulu University Hospital, Finland; 4 Research Unit of Population Health, University of Oulu, Finland

**Keywords:** SMI, STAX-SA, schizophrenia, outcome, rehabilitation, supported living, supported housing

## Abstract

**Objective:**

The transition from institutional psychiatric care to community-based mental health services has resulted in the rapid development of assisted living services (AL) for mentally ill. Focus of the current study is to add internationally comparable evidence-based knowledge on the rehabilitation of AL residents by examining progression and mortality in relation to the level of service provided in AL units.

**Methods:**

This study utilized data gathered from a longitudinal study conducted in Finland during the years 2020 to 2022. A total of 340 health- and social care records of residents in AL services were examined over a 3-year study period. Progression (changes in AL service level) and mortality of AL residents were explored through the level of service provided in AL units, by applying the Simple Taxonomy for Supported Accommodation (STAX-SA). Progression was grouped into (1) progressed, (2) stable, and (3) regressed.

**Results:**

During the 3-year study period progression was examined for 95.3% (*n* = 324) of the AL residents of which 18.8% progressed into less supported AL, 79.3% remained stable and 1.9% regressed into more supported AL. In the entire population (*n* = 340) mortality was 4.7%.

**Conclusion:**

AL residents progressed into less supported AL services rarely although they had similarities in clinical characteristics. This might indicate that the development of AL services has evolved into a more custodial type rather than rehabilitation. Progress of rehabilitation in AL services should be investigated further by examining AL resident characteristics in relation to the level of service and level of support provided in AL services.

## Introduction

Since the 1980s psychiatric services, both globally and in Finland, have undergone a major change in which patients with severe mental illness (SMI) have been attempted to integrate into society (R. K. R. Salokangas & Saarinen, 1998). This has required the rapid development of different types of community-based services away from asylums (Chilvers et al., 2002; Honkonen et al., 1999; Jahangiri et al., 2022; R. Salokangas et al., 2000; Tuori et al., 1998). This change has transformed the focus of care from long-term psychiatric inpatient treatment into outpatient care services, supported by a network of different housing services named assisted living services (AL; Jahangiri et al., 2022). This transition has emphasized AL as a crucial part of psychiatric care and rehabilitation services (Ketola et al., 2021). AL services for SMI have had an impact in reducing psychiatric inpatient treatment use and costs (Broulíková et al., 2019; H. Chan et al., 2007; Killaspy et al., 2020; Rog, 2004). Some studies have reported that AL services can provide proper rehabilitation with adequate outcomes, such as reduction in psychiatric symptoms and increased quality of life (Tolonen et al., 2021, 2024). However, these studies have not been able to analyse the level of services provided in AL units in relation to outcome measures of their residents, thus a need for longitudinal research on the effectiveness of AL services has been emphasized (Chilvers et al., 2006; Ketola et al., 2021; Killaspy & Priebe, 2021). Due to the lack of global taxonomy for different AL services and inconsistent reporting of AL units and residents made the national and international evaluation and comparison of AL services challenging (Jahangiri et al., 2023; Killaspy & Priebe, 2021; McPherson et al., 2018).

We conducted a register-based research project, named the Porvoo ASPA-project in Finland, aiming to enable the longitudinal evaluation of effectiveness in AL services in Finland (Jahangiri et al., 2023). The focus of the current study is also to add internationally comparable evidence-based knowledge on the rehabilitation of AL residents by examining progression and mortality as outcome measures in relation to the level of service provided in AL units. We utilized the international and structured “The Simple Taxonomy for Supported Accommodation” (STAX-SA types) taxonomy for service level categorisation of AL units and the data collected during a 3-year follow-up period (Jahangiri et al., 2023; McPherson et al., 2018).

## Aims

This study aims to evaluate the outcome (progression and mortality) of AL residents in terms of their sociodemographic and clinical characteristics and the types of STAX-SA of the AL units they lived in.

## Materials and methods

This study examined the individual-level data (i.e. age, gender, medication and diagnoses) of residents living in AL units for mentally ill. A description of the study procedure is presented in an earlier publication (Jahangiri et al., 2023). The data was collected from the electronic health and social care records of the city of Porvoo. The STAX-SA was used for AL service categorisation in this study (Jahangiri et al., 2023; McPherson et al., 2018).

The STAX-SA is a domain-based categorization for AL services developed for categorizing AL services for people with mental disorders. It comprises five types of supported housing, which are identified by classifying services based on four domains (staffing location, level of support, emphasis on move-on and physical structure; McPherson et al., 2018). The level of services and support gradually decrease from STAX-SA 1 (staff on-site, limited emphasis on move-on with high level of support in a congregate setting) providing the highest level of services and support to STAX-SA 4 (no staff on-site with low level of support in a individual accommodation setting) with the lowest level. The STAX-SA taxonomy differentiates AL units also on how much emphasis is focused on residents’ progression to less supported AL services (McPherson et al., 2018). In Finland, the AL service system does not include STAX-SA 5 because there is no such service where staff is on-site with no support (Jahangiri et al., 2023).

## Study population

**Figure 1.**
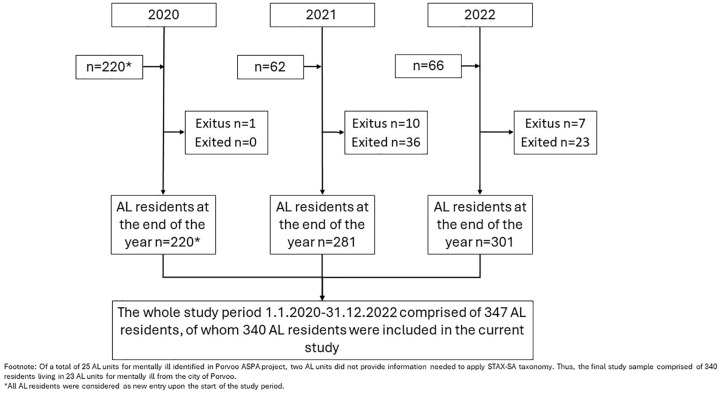
The flow chart for sample selection.

The individual-level data of AL residents consisted of adults aged 18 years or above from the city of Porvoo, and who had resided in AL units for mentally ill, based on the social welfare decision for AL, during the 3-year study period from 1.1.2020 to 31.12.2022. The final study population used in the statistical analyses comprised of AL residents (*n* = 340) whose AL units had provided enough information to enable categorisation of their service level according to STAX-SA taxonomy (Jahangiri et al., 2023). The flow chart for sample selection process is presented in Figure 1.

The variables from individual-level data used in this study are described in Appendix 1. The psychiatric diagnoses were grouped according to the International Classification of Diseases, version 10 (ICD-10; World Health Organization, 1993). The psychotropic medication was categorized by the Anatomical, Therapeutical and Chemical Classification System (ATC) for the nervous system (ATC-N; World Health Organisation, n.d.).

## Outcome measures

Outcome measures used in this study were progression and mortality of AL residents. Progression outcome was defined based on the change in STAX-SA type of AL unit of a resident between entry to the study and the end of the study period. It was grouped into three categories: (1) Progressed group consisted of AL residents, who moved-on to less supported AL services (STAX-SA type increased) or who exited totally from AL services, (2) Stable group included AL residents, whose STAX-SA type did not change upon entry and end of the study period and (3) Regressed group (meaning the residents need for services increased) comprised of AL residents living in a more supported AL service than at the entry to the study (STAX-SA type decreased).

## Statistical analyses

The sociodemographic and clinical characteristic of AL residents were compared in relation to STAX-SA types (1–4) and progression of AL residents. Statistical significance of group differences in categorical variables was analyzed with Pearson Chi-square test or Fisher’s exact test and in continuous variables with Student’s-test or ANOVA-test. A Sankey diagram was utilized to illustrate AL residents’ path concerning outcome categories for progression (progressed, stable, regressed) and mortality (Lamer et al., 2020). The statistical software used in analysis was IBM SPSS statistics, version 29.

## Results

### AL resident sociodemographic and clinical characteristics according to STAX-SA

As Table 1 presents, in the total data (*n* = 340) of AL residents, the mean age was 47 years (SD 16.2) and majority were males (62.4%). Age of AL residents differed between STAX-SA types (*p* < .001), from the oldest with on average 60 (SD 13.7) years living in STAX-SA 1 youngest in STAX-SA 4 being 45 (SD 15.6) years of age. Gender distribution of AL residents did not differ statically significantly between the types of STAX-SA.

**Table 1.** Sociodemographic and clinical characteristics of AL residents, by the types of STAX-SA.

	The types of STAX-SA										*p*-Value*
	Total		STAX-SA 1		STAX-SA 2		STAX-SA 3		STAX-SA 4		
Variables	*N*	%	*N*	%	*N*	%	*N*	%	*N*	%	
Gender											
Males	212	62.4	13	52.0	28	52.8	14	66.7	157	65.2	.245
Mortality	16	4.7	5	20	4	7.5	0	0	7	2.9	<.004^a^
Any psychiatric disorders	216	63.5	15	60.0	33	62.3	12	57.1	156	64.7	.875
Major psychiatric disorder											
Schizophrenia	45	13.2	6	24.0	15	28.3	6	28.6	18	7.4	<.001
Psychosis	23	6.8	4	16.0	7	13.2	1	4.8	11	4.5	
Affective disorders	42	12.4	1	4.0	5	9.4	2	9.5	34	14.1	
Substance use-related disorders	79	23.2	4	16.0	4	7.5	2	9.5	69	28.6	
Other mental disorders	27	7.9	0	0	2	3.8	1	4.8	24	10.0	
Not recorded	124	36.5	10	40.0	20	37.8	9	42.8	85	35.2	
Comorbid diagnosis	118	34.7	6	24.0	17	32.1	5	23.8	90	37.3	.352
Most common comorbid diagnostic groups											
Schizophrenia + substance	7	2.1	0	0	2	3.8	1	4.8	4	1.7	.353
Psychosis + substance	7	2.1	1	4.0	1	1.9	0	0	5	2.1	.770
Affective + substance	12	3.5	0	0	0	0	2	9.5	10	4.2	.154
Psychotropic medication	190	55.9	12	48.0	31	58.5	16	76.2	131	54.4	.211
Most common psychotropic medication											
Oral antipsychotic	100	29.4	9	36.0	26	49.1	13	61.9	52	21.6	< .001
Long-acting injectable (LAI)	12	3.5	1	4.0	2	3.8	4	19.1	5	2.1	.007
Mood stabilizers	39	11.8	1	4.0	11	20.8	4	19.1	23	9.5	.046
Depression medication	106	31.2	6	24.0	13	24.5	5	23.8	82	34.0	.371
BNZ* medication	81	23.8	5	20.0	17	32.1	7	33.3	52	21.6	.272
Previous living arrangements											
Homeless	6	1.8	1	4.0	0	0	0	0	5	2.1	ne
Home	29	8.5	2	8.0	11	20.1	7	33.3	9	3.7	
Home with supported living services	28	8.2	3	12.0	25	47.1	0	0	0	0	
AL service with supervision	13	3.8	3	12.0	3	5.7	6	28.6	1	0.4	
Not known	264	77.5	16	64.0	14	26.4	8	38.0	226	93.8	
Main sources of income											
Pension	167	49.1	22	88.0	49	92.5	15	71.4	81	33.6	< .001
Other source of income	77	22.6	3	12.0	4	7.5	5	23.8	65	26.9	
Not known	96	28.2	0	0	0	0	1	4.8	95	39.4	

*Note*. The amount of missing data per variable per STAX-SA types varies due to lack of such information in data sources available for our study. For example, percentage of missing data was notably high in the STAX-SA 4 (*n* = 241) in variable for the previous living (93.8%). Ne = not justifiable to estimate due to high proportion of missing information; *SD* = standard deviation, BNZ = benzodiazepine; AL = assisted living.

a Fisher-Freeman-Halton Exact Test.

* Group difference *p*-value.

As seen in Table 1, the psychiatric diagnosis could be identified from data sources for 63.5% of all AL residents. Major psychiatric disorders differed significantly between STAX-SA types (*p* < 0.001). Diagnosis for schizophrenia prevailed in STAX-SA 1 (24%), STAX-SA 2 (28.3%) and STAX-SA 3 (28.6%), while affective disorders were common in STAX-SA 4 (14.1%). Diagnosis for substance abuse was a characteristic for residents in STAX-SA 1 (16%) and STAX-SA 4 (28.6%). Diagnostic information was lacking 36.5% of the AL residents. Comorbidity of diagnoses did not statistically significantly differ between the STAX-SA types.

The use of psychotropic medication was reported for 55.9% of AL residents, the most common medication groups being depression medication (31.2%), BNZs (23.8%). The use of oral antipsychotics differed significantly between STAX-SA types (*p* < .001), varying from 61.9% in STAX-SA 3 to 21.6% in STAX-SA 4. Long-lasting injectables (LAIs) were more prevalent in STAX-SA 3 (19.1%) compared to other STAX-SA types (range from 2.1% to 4.0%) (*p* = .007). Use of mood stabilized was more common in STAX-SA 2 (20.8%) and in STAX-SA 4 (19.1) compared to other STAX-SA types (range from 4.0% to 9.5%) (*p* = 0.046).

Previous living arrangements such as homeless, home or living in different types of AL services were reported only for 22.5% of AL residents. Of those AL residents, whose previous living arrangement was known, home with supported living services and AL services with supervision prevailed in the STAX-SA 1 type, home and home with supported living services in STAX-SA 2 type, and home and AL services with supervision in STAX-SA 3. Main source of income was reported for 71.2% of AL residents. Pension as main source of income prevailed in STAX-SA types 1, 2, and 3 (*p* < .001).

### AL residents’ outcome (progression and mortality)

**Figure 2.**
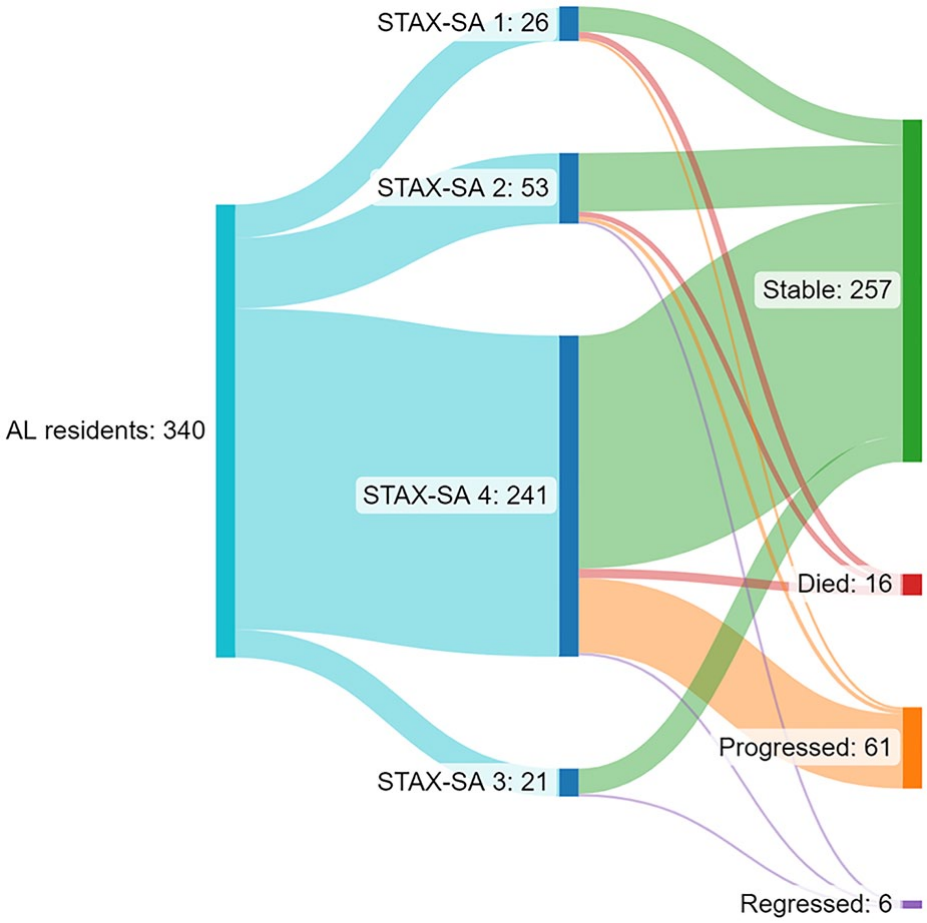
Sankey illustrates the distribution of different outcome groups of the study population.

Figure 2 illustrates with the Sankey diagram the progression and mortality rates as outcomes of AL residents. Progression outcomes were evaluated for 324 AL residents, who were alive over the whole three-year study period. Of them, a total of 18.8% (*n* = 61) progressed to less supported AL services, 79.3% (*n* = 257) remained stable and 1.9% (*n* = 6) regressed to more supported AL services. The mortality of AL residents was 4.7% (*n* = 16). Of them 81.3% (*n* = 12) were males and their mean age was 63 (SD 9.98) years. Of the 16 who died represented 20% in STAX-SA 1 (mean age 55 SD 10.01), 7,5% in STAX-SA 2 (mean age 66 SD 7.53) and 2.9% in STAX-SA 4 (mean age 54 SD 11.10) (*p* < .004).

Table 2 shows progression of AL residents in terms of their sociodemographic and clinical characteristics. Of all 215 AL residents with information of psychiatric disorder 18.6% progressed, 78.6% stable and 2.8% regressed group, the corresponding proportions in AL residents without a recorded psychiatric disorder being 19.3% progressed, 80.7% stable and none in regressed group. In that group, youngest AL residents were in progressed (mean 40.4 SD 14.6) and oldest in regressed group (mean 56.8, SD 12.2) (*p* = .033). Further, comorbid diagnoses differed between the progressed (57.5%), stable (53.9%) and regressed (66.7%) group (<.001) as well as the use of oral antipsychotics differed between progressed (15%), stable (37.9%) and regressed (83.3%) groups (<.001).

**Table 2.** Progression of AL residents during the 3-year study period.

	Total^a^		Progression											
			Psychiatric disorder reported							Psychiatric disorder not recorded				
			Progressed		Stable		Regressed		*p*-Value*	Progressed		Stable		*p*-Value*
Variables	*N*	%	*N*	%	*N*	%	*N*	%		*N*	%	*N*	%	
Gender														
Males	199	61.4	25	62.5	105	62.1	3	50	.867	15	71.4	51	58	.324
STAX-SA type														
STAX-SA 1	21	6.48	2	5.0	13	7.7	0	0	.012	–	–	6	6.8	.050
STAX-SA 2	49	15.1	2	5.0	28	16.6	2	33.3		1	4.8	16	18.2	
STAX-SA 3	21	6.48	0	0	10	5.9	2	33.3		–	–	9	10.2	
STAX-SA 4	233	71.9	36	90	118	69.2	2	33.3		20	95.2	57	64.8	
Any psychiatric disorder	215	66.4	40	100	169	100	6	100	–	–	–	–	–	–
Major psychiatric disorder														
Schizophrenia	45	13.9	7	17.5	35	20.7	3	50	.645	–	–	–	–	–
Psychosis	23	7.1	4	10.0	18	10.7	1	16.7		–	–	–	–	
Affective disorders	41	12.7	10	25.0	31	18.3	0	0		–	–	–	–	
Substance use related disorders	79	24.4	13	32.5	64	37.9	2	33.3		–	–	–	–	
Other mental disorders	27	8.3	6	15.0	21	12.4	0	0		–	–	–	–	
Not recorded	109	33.6	–	–	–	–	0	0	–	–	–	–	–	–
Comorbid diagnosis	118	36.4	23	57.5	91	53.9	4	66.7	<.001	–		–	–	–
Most common comorbid diagnostic groups														
Schizophrenia + substance	7	2.2	1	2.5	6	3.6	0	0	1.000	–	–	–	–	–
Psychosis + substance	7	2.2	3	7.5	4	2.4	0	0	.288	–	–	–	–	–
Affective + substance	12	3.7	3	7.5	9	5.3	0	0	.791	–	–	–	–	–
Psychotropic medication	189	58.3	21	52.5	115	68.0	6	100	.039	9	42.9	38	43.2	1.000
Most common psychotropic medication														
Oral antipsychotic	100	30.9	6	15.0	64	37.9	5	83.3	<.001	–	–	25	28.4	.007
Long acting injectable (LAI)	12	3.7	–	–	6	3.6	2	33.3	.007	1	4.7	3	3.4	1.000
Mood stabilizers	39	12.0	2	5.0	24	14.2	1	16.7	.290	2	9.5	10	11.4	1.000
Depression medication	105	32.4	16	40.0	65	38.5	2	33.3	1.000	8	38.1	14	15.9	.034
BNZ medication	80	24.7	9	22.5	48	28.4	4	66.7	.085	5	23.8	14	15.9	.521
Previous living arrangements														
Homeless	6	1.9	1	2.5	5	3.0	0	0	.058	–	–	–	–	.138
Home	25	7.7	1	2.5	14	8.3	2	33.3		2	9.5	6	6.8	
Supported living services	4	1.2	1	2.5	2	1.2	1	16.7		–	–	–	–	
AL service with supervision	35	10.8	3	7.5	16	9.5	1	16.7		–	–	15	17.1	
Not known	254	78.4	34	85.0	132	78.1	2	33.3		19	90.5	67	76.1	
Main source of income														
Pension	153	47.2	21	52.5	70	41.4	3	50.0	<.001	12	57.1	47	53.4	.086
Other	75	23.1	19	47.5	37	21.9	2	33.3		6	28.6	11	12.5	
Not known	96	29.6	–	–	62	36.7	1	16.7		3	14.3	30	34.1	

*Note*. There were no regression in psychiatric disorder not recorded group. There were no homeless or living in supported living services either. *SD* = standard deviation; BNZ = benzodiazepine; AL = assisted living.

a Excluding those AL residents that died n = 324.

* Group difference p-value.

## Discussion

This study aimed to examine the rehabilitation of residents in AL services for mentally ill by evaluating the progression longitudinally over a study of 3 years. Change in STAX-SA type categorisation level of services was used as an indicator for progression, which has not been used, to the best of our knowledge, so far.

Our main finding was that approximately only one in five AL residents progressed into less supported AL services regardless of having a recognized psychiatric disorder or not.

In Finland AL services for mentally ill were developed for individuals suffering from SMI, schizophrenia in particular, to cope in the community instead of living in custodial-type psychiatric facilities (Tuori et al., 1998). In our study, the diagnosis for psychiatric disorders was recognized for 66.4% of AL residents, only 18.6% of them progressed into less supported AL services during the study period (Killaspy & Priebe, 2021; Salisbury et al., 2017). Only 31% of residents with a diagnosis suffered from psychotic disorders. These numbers can be considered substantially low when compared to previous studies. For example, in earlier studies from the UK and Finland, 23% to 78% of AL residents have shown to progress to less supported AL services although in these studies populations have consisted of residents with some SMI diagnosis (K. P. K. Chan et al., 2021; Killaspy & Zis, 2013; Tolonen et al., 2022). Our findings, considering the progress in rehabilitation, were in line with the findings of K. P. K. Chan et al. (2021) 23% (follow-up 5 years) but still low compared to Killaspy and Zis (2012) 40% (follow-up 5 years) and substantially lower than Tolonen et al. (2022) 78% (follow-up 7 years; K. P. K. Chan et al., 2021; Killaspy & Zis, 2013; Tolonen et al., 2022). One plausible explanation for this difference might be the shorter follow-up (3 years) in our study, which might indicate that this time frame has been too short for the progression of rehabilitation. However, it is also reasonable to question the rehabilitative ability of AL services, as the progression of AL residents appears not to take place to the extent, that might be assumed for SMI according to earlier studies (Harvey et al., 2023).

In our study, psychotic disorders were equally prevalent in all STAX-SA types. This finding was surprising because it differs from international studies, where most of the psychotic disorders were concentrated in units with high levels of support (STAX-SA 1 and 2) (Ketola et al., 2021). Further, according to our finding’s progression was more common among AL residents in less supported AL services (STAX-SA 4) contradicting international studies where most progress was from heavily supported AL services (STAX-SA 1 and 2) (K. P. K. Chan et al., 2021; Ketola et al., 2021). In the study of K. P. K. Chan et al. (2021) progression was more common among residents in heavily supported AL services than among those in less supported AL services. In our study, there was also a surprisingly low rate of regression of only 1.8% compared to numbers of the study by Killaspy & Zis (2013) (38%). This difference can be at least partially explained because in their study psychiatric inpatient treatment use was seen as regression, which we were not able to analyse (Killaspy & Zis, 2013).

In this study social- and healthcare electronic patient registers were used to ensure a multidisciplinary approach to data collection considering diagnostic information. However, the proportion of AL residents, without information of diagnosis for psychiatric disorders, was substantial and concentrated in less supported AL services (STAX-SA 4). This finding is interesting and raises many questions, considering that the population of this study consisted of AL residents living in AL services for individuals with mental illnesses. One explanation might be the Social Welfare Act that governs AL services in Finland, not requiring a psychiatric diagnosis for entering AL services (Sosiaalihuolto Laki 30.12.2014/1301, 2014). Also, the nationally approved Housing First model in Finland might be one reason. According to this model, housing, when needed, is acquired for an individual regardless of the diagnoses (Shinn & Khadduri, 2020). Substance abuse might explain the absence of diagnoses as very often they are not assessed in health care and are often considered more social problems than health-related ones. This is considered problematic in the Finnish service system where substance-related problems are mainly cared for and assessed by the social welfare sector without the support of health care professionals (Sosiaalihuolto Laki 30.12.2014/1301, 2014). An individual might be left out of the healthcare system in general and not receive the necessary healthcare services. The lack of diagnostic data, on the other hand, may indicate that AL residents’ potential need for adequate and active treatment and rehabilitation plans does not occur. Therefore, the population in AL services without a psychiatric disorder should be studied further to ensure that the necessary rehabilitation they need is based on equity not solely equality. This might also clarify what types of housing services are possibly missing in the current service system, and thus ensure the prevention of possible misuse of AL services for the mentally ill in Finland.

In earlier studies, the mortality rate of AL residents is reported to be higher than that of the general population. In our study mortality rate was 4.7% (Nordentoft et al., 2012), being lower compared to the UK (12%) and Denmark (21%) (Killaspy & Zis, 2013; Nordentoft et al., 2012). In our study, 56% of the AL residents that died, lived in heavily supported AL services (STAX-SA 1 and 2). In STAX-SA 1 mortality rate was 20% and in STAX-SA 2 it was 7.5%. Assumably those AL residents in heavily supported AL services (STAX-SA 1 and 2) are older and thus in need of more support and care, also for physical reasons, because of the high prevalence of comorbid somatic health issues. Although mortality rates can be considered low, it is important to understand that AL services often employ non-healthcare professionals without capability to intervene to somatic issues on time. Therefore, the somatic issues of residents should also be studied further and be an active part of the service system.

The use of oral antipsychotics increased from STAX-SA 1 to 3 but was the least in STAX-SA 4. This finding is quite reasonable considering that antipsychotic medication is mostly used for SMI who also need intensive and high-level support. From an outcome perspective, the regression group also had more oral antipsychotic use, and 1/3 of the group had long-acting injectable (LAI) medication emphasizing the long-lasting need of support for SMI. Interestingly the use of LAI was low in general and there was no difference between STAX-SA type or outcome group in LAI use. It would have been expected to have more LAI use in the progressed group as active use of psychotropic medication is widely known to reduce relapses (Munday et al., 2019).

In our previous study, we identified the growth of STAX-SA 4 level services compared to STAX-SA 1, 2 and 3, being in line with this study where most AL residents were in STAX-SA 4 (Jahangiri et al., 2022). Based on our previous studies we argued that the lack of other mental health services leads to the inappropriate use of AL services. This seems to occur as mental health professionals and individuals in need of mental health services are forced to rely on AL services, which are not resourced enough to adequately conduct psychiatric assessments, care, and rehabilitation (Jahangiri et al., 2022). This seems to be the case as the rates of progression in this study remained low. Therefore, it is assumable that the role of AL services has been shifting to more custodial care instead of active rehabilitation as a part of planned treatment. This direction of development has been possible due to the slow growth of outpatient care services, which was shown in our previous study, and AL services are not able to respond to the need for treatment (Jahangiri et al., 2022).

## Conclusion

AL services for the mentally ill in Finland seem to consist of a clinically heterogeneous population of residents. There appears to be residents without a psychiatric disorder but are for some reason in need of AL services for mentally ill. Taking into consideration the low rate of progression in heavily supported AL units it seems that AL services are transferring back into the era of institutionalization and evolving into a more custodial type of care rather than rehabilitation of residents. It is crucial to examine the rehabilitation of residents taking place in AL units in a multidisciplinary and longitudinal manner by including psychiatric inpatient treatment records, as well as other outcome measures such as functioning and quality of life. This approach would enable the proper examination of the effectiveness of AL services for rehabilitation.

## Limitations and strengths

The methodological strengths of this study were the longitudinal comparative design (STAXA-SA types) and the use of information from both electronic health- and social care registers. The major limitation of this study was amount and heterogeneity of the data available for our study purpose, particularly, a lot of information of residents receiving supported living services (STAX-SA 4) were not available from the register-based data sources. The replacement of missing data with applying some statistical approach was considered likely to produce vague information about resident’s true characteristics and service needs. Further, this study was unable to examine whether use of AL services had reduced need for psychiatric inpatient hospitalization, because the data on psychiatric hospital admissions was not available for research purposes. Unfortunately, it was not possible to calculate for all residents the total length of stay in AL services, since we did not have access on data sources beyond our data collection period. Our research permissions covered the period from 1.1.2020 to 31.12.2022, and no access on AL service data prior 1.1.2020 or after 31.12.2022 was granted. The only available information, which was utilized to identify our study population, was the date when the decision for granting access (entry) to AL service was made. This date of decision commonly precedes the actual date of entry to AL services. This study was conducted during the COVID-19 pandemic. It was not possible to evaluate its effects on the study findings.

## Appendix

**Appendix 1.** Study variables used for this study.

Variable	Description
Psychiatric diagnoses	ICD-10^1^ codes
Schizophrenia	F20.x
Psychosis	F21.x-F29.x
Affective disorders	F31.x-F39.x
Substance abuse disorder	F10.x-F19.x
Other mental disorders	All other F-coded diagnoses
Psychiatric comorbidity	Any two F-coded diagnoses occurring simultaneously
Psychotropic medication	ATC-N^2^ codes
Oral antipsychotics	N05A
Long-acting injectable antipsychotics (LAI)	N05A
Mood stabilizers	N03A
Antidepressants	N06A, N06C
Benzodiazepines or related drugs	N05B, N05C
Prior housing arrangements	
Homeless	No prior living arrangement
Home	Living alone or living with family
Home with supported living services	Living alone with mental health-specific services provided on- or off-site
AL service with supervision	Living in a mental health-specific AL service with 24-hour or part-time service provided on-site
Source of income	
Pension	Any type of pension
Other benefits	Salary and student benefits
Unemployment benefits	Unemployment payments

*Note*. AL = assisted living; ICD-10 = international classification of diseases, version 10; ATC-N = Anatomical, Therapeutical and Chemical Classification System (ATC) for the nervous system.
